# Supplementary material for: Health-related quality of life and associated factors among patients with stroke at tertiary level hospitals in Ethiopia
Source: PLoS One. 2021 Mar 18;16(3):e0248481. doi: 10.1371/journal.pone.0248481 (PMC7971497; doi:10.1371/journal.pone.0248481)
Supplement: Attachment — Submitted filename: PONE-D-20-19104.docx [file pone.0248481.s002.docx]

Dear author(s),

Many thanks for choosing this subject. I appreciate you because of all efforts, short of some recommendation below:

1. In your abstract, please do not change the subtitles. I mean Purpose. Write introduction instead of Purpose.
2. In abstract, please mention to the statistical population and the sample and the method of sampling. It is vital that author(s) present the area I which the study was carried out.
3. Your results in you abstract needs substantial changes. Please follow the routine methods to write this part. It is incomplete and the SD and p-value should be given where the estimations are reported.
4. There are a few keywords please extract further related keywords from <https://www.ncbi.nlm.nih.gov/mesh>.
5. About your introduction, please elaborate a little bit on HRQOL in the other areas and the importance of this subject. Some parts of Introduction part are related to Discussion part, so please transform them into Discussion part.
6. Why was proportional sampling chosen? And please state the type of sampling.
7. Is there any eligibility criteria? Please cite to related references where the inclusion and exclusion are confirmed.
8. Why was semi-structured interview opted? These types of interviews likely increase the personal bias. Please explain whether this conduction is solo or not.
9. Why was consecutive sampling done while your sampling is proportional? Bias can also occur in consecutive sampling when consecutive samples have some common similarity and it is a non-probability sampling.
10. Your instrument part is too long. To avoid prolixity, please explicate the main items briefly and cite to most related manuscript which can confirm what have been done.
11. Were the presumptions of ANOVA and multiple regression models checked?
12. Was there any outlier or influential observation in your data?
13. Was there any multicollinearity among main variable?
14. Why is a nominal p-value considered before modelling? Is there any valid source to cite? What is the type of model selection? Which kinds of model adequacy checking criteria were chosen?
15. What is the ground to separate two models for physical and mental component? Is there any previous knowledge to do that?
16. Please follow the general writing rules to write the figures. Write the figures with equal number of decimal.
17. Generalized linear model (GLM) is broadly used to analyze data in which there are some categorical predictors or covariates. Was this approach applied? This approach is different from multiple linear regression models, so please state the correct method.
18. Please interpret the effect of predictors clearly and completely and is there any moderator effect upon the related response variable?
19. Why was cut-off considered for some of covariates, for instance, income and education? You definitely lost a part of information by categorizing the continuous variables.
20. You have checked the relations between predictors and response variables and response comprises of 3 components. How did you examine the effects of each component? Which component is the origin of effect in each model? How you convince these parts of effects?
21. There is not any plot or path diagram which can illustrate the path between variables. Moreover, there is not any plot to for descriptive analysis. It is a plus point for your manuscript to display the effects schematically.

With regard to these comments, I thoroughly recommend author(s) revise their paper majorly.

Regards,

Maryam Ganji
