# Supplementary material for: Health-related quality of life and associated factors among patients with stroke at tertiary level hospitals in Ethiopia
Source: PLoS One. 2021 Mar 18;16(3):e0248481. doi: 10.1371/journal.pone.0248481 (PMC7971497; doi:10.1371/journal.pone.0248481)
Supplement: Attachment — Submitted filename: Response to Reviewers.docx [file pone.0248481.s003.docx]

**Date: January 5, 2021**

**Subject**: **Response to reviewers**

**Manuscript title: Health-related quality of life and associated factors among patients with stroke at tertiary level hospitals in Ethiopia**

**Manuscript ID: PONE-D-20-19104**

Dear Anandh Babu PonVelayutham,

We appreciate the efforts by you and the reviewer on the manuscript. We thank you and the reviewer for the thorough reading and constructive comments and questions of our manuscript and for the opportunity to revise and resubmit. We are pleased to submit the improved research article, including language editing and formatting according to the journal requirement, “Health-related quality of life and associated factors among patients with stroke at tertiary level hospitals in Ethiopia” for your consideration in PLOS ONE. On the following pages, you will find our response to reviewer comments. On behalf of my co-authors, I thank you for your consideration of this resubmission. We appreciate your time and look forward to your response.

**Response to Reviewer**

Dear Maryam Ganji,

Thank you very much for your comprehensive comments and constructive suggestions. We read and consider each comment very carefully, and thoroughly revise the manuscript according to your comments and suggestions. We have also edited the language. We hope that the manuscript reads more convincingly after the revision.

**Reviewer #1: Dear author(s),**
1. Your abstract is now long, please shorten but do not omit the main part as I said before. In your results part in your abstract, it is dispensable to state all results, just mention to them but with those cautions that I mentioned previously.

**Response:**

Dear, thank you for the comments, we have revised the abstract to shorten it without losing the main part. Please, see the revised manuscript.

2. In your data analysis part, please explicate why p-value <0.2 was chosen or please present an evidence about that. Please cite to a reference.

**Response:**

Thank you very much for the comment. There is a debate about the use of p-value for variable selection. However, often arbitrary choice has to be made about the selection parameter, that is, the significance level to decide whether an effect should be retained in a model. One of the ways to do that is to screen the variables by using p-value such as P value of 0.2 in the bi-variable analysis.

*Evidence:* *Heinze G, Dunkler D. Five myths about variable selection. Transplant International. 2017;30(1):6-10.* This reference has been cited in the data analysis part. Please, see reference number 42.

The main reason why we use the p-value to select variables is to have a parsimonious model, which is a model with the smallest number of variables that have a high effect on the outcome variable and this has its contribution to finding better estimates. The probability of the variables that have a p-value >0.2 from the bi-variable analysis to be significant on the final multivariable model is almost zero. Therefore, we believe adding those variables to the model may affect the parsimony and may also cause overfitting of the model. Moreover,it is usually advised that not just one criterion should be used but if possible an array of criteria. Common model selection criteria are R^2^, AIC, BIC, and p-level. Therefore, we have used p-value and AIC criteria to get the best final model.

Finally, as we mentioned in the previous response, usage of P value <0.2, during bivariable analysis for variable selection is common practice in our setup.

3. Please state the presumptions of ANOVA models and check them.

**Response:**

Thank you! There are six "assumptions" that underpin the one-way ANOVA. Since the first three assumptions (d**ependent variable** should be measured at the **continuous level**, the **independent variable** should consist of **two or more categorical, independent (unrelated) groups,** and **independence of observations)** cannot be tested for using software,  **we have addressed them during** study design and choice of variables**.** But, we can check the rest three assumptions: **no significant outliers. dependent variable** should be **approximately normally distributed, and homogeneity of variances**. We have mentioned the three assumptions in the method part of data analysis, and detailed results of the assumptions were given in the result section. Please, see line numbers 132-133 and 181-187.

4. Please give a reason for making the covariates (income and education level) categorical. It helps you to enhance the validity of your manuscript.

**Response:**

Dear, thank you for your comments. As we mentioned in the previous response, when the literature review was done, those variables, in most literature, were found as categorical, and for direct comparison purposes, we developed the questionnaire as a closed question (categorical). Unfortunately, we are unable to change those variables into continuous like social support and depression.

5. Please make your tables shipshape interms of subheadings. (table3and4)

**Response:**

Thanks for pointing out our shortcomings! We have revised the subheadings of Tables 1, 3 & 4.

6. It would be very very nice if you interpreted the results clearly and please do not confine your paper to just reporting. It will be very nice if you report the measure of effect of each predictor.

**Response:**

Dear, thank you for your constructive comments. We have interpreted each coefficient, which was significantly associated with each domain of quality of life. Please, see line numbers 199-207 and 215-220.

7. Please write a subtitle for your figure. It is vague. It is better than the last version but it still needs to revise more.

**Response:**

Thank you for your suggestions! we have revised the figure such as subtitle. Please see the revised figure.
